# A Rare Case of Bacteremic Community-Acquired Pneumonia Due to Pasteurella Multocida Presenting With Hemoptysis

**DOI:** 10.7759/cureus.14232

**Published:** 2021-03-31

**Authors:** Mohamad F Ayas, Dima Youssef, Leonard Johnson

**Affiliations:** 1 Internal Medicine, Ascension St. John Hospital, Detroit, USA; 2 Infectious Diseases, Ascension St. John Hospital, Detroit, USA

**Keywords:** pasteurella multocida, community aquired pneumonia, hemoptysis

## Abstract

*Pasteurella multocida* (PM) is a gram-negative bacterium known to cause soft tissue infections, especially after animal bites, with some human infections occurring after animal exposure, usually via inhalation of contaminated secretions. PM pneumonia mainly affects those who are immunocompromised and in individuals with comorbidities. The spectrum of pulmonary disease due to PM is wide, ranging from pneumonia to empyema. The clinical features are indistinguishable from other pathogens, however, hemoptysis seldom occurs as a consequence of PM infection. We present a case of PM pneumonia in an immunocompetent host who had a chief complaint of hemoptysis, making this the sixth documented case to ever-present with hemoptysis.

## Introduction

*Pasteurella multocida* (PM) is a gram-negative coccobacillus that colonizes the oropharynx and gastrointestinal tract of many wild and domestic mammals especially cats and dogs [1,2]. The most common human infections reported and known are those of soft tissue and skin infections from animal bites and scratches [2]. In patients with underlying lung disease, it can cause a variety of upper and lower respiratory tract infections, as PM also colonizes the respiratory tract [3]. Although mainly transmitted through direct animal contact, it was found that about 16-31% have no known animal contact contributing to their infection [4]. It was proposed that those infections develop in the nasopharynx or other upper respiratory tract mucosa through contact with animals or animal secretions or through inhalation with later dissemination [4]. While most cases present with typical signs of pneumonia, we report a patient presenting with hemoptysis.

## Case presentation

**Figure 1:**
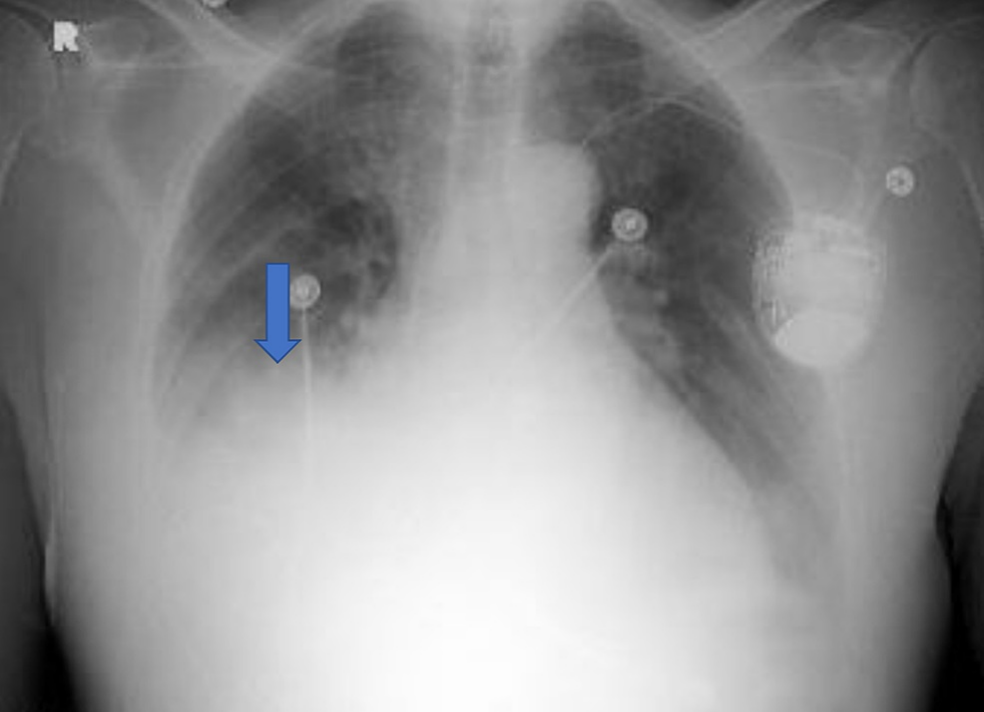
Chest X-Ray Revealing a Right Middle Lobe Infiltrate (Blue Arrow).

**Figure 2:**
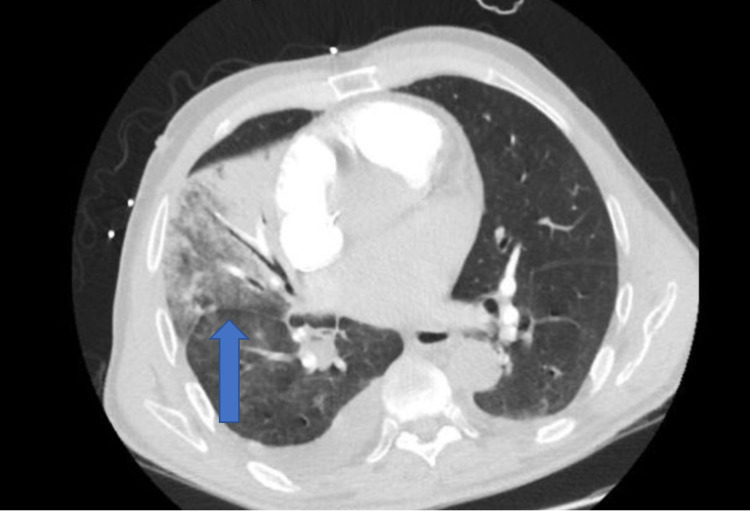
CTA of the Chest Revealing Right Pulmonary Infiltration in the Right/Middle Lobe With Basilar Parapneumonic Effusion (Blue Arrow). CTA: computed tomography angiography

A 79-year-old male with a past medical history of atrial fibrillation on apixaban, congestive heart failure, and hypertension presented to the emergency department with a one-day history of hemoptysis, shortness of breath, diarrhea, and chest pain. The patient was in his usual state of health until the day before where he started coughing up blood. Four days prior to admission, he sustained a dog bite to his hand that didn’t require medical attention. He stated that he had a subjective fever, chills, difficulty breathing, headache, and had three watery bowel movements a day. He has tested one year ago for tuberculosis (TB) and it was negative. Furthermore, he denied any travel history or sick contacts. On admission, the patient had a blood pressure of 83/53 mmHg, respiratory rate of 21 breaths/minute, oxygen saturation of 93% on room air, and had a heart rate of 114 beats/minute. On examination, the patient was extremely lethargic, the lung examination showed rhonchi in the right middle and lower lobes with diminished breath sounds at bases bilaterally. There were no bite marks or other skin findings. Initial laboratory findings showed a white blood cell count of 15.9 K/mcL, lactic acid of 8 mmol/L, C-reactive protein of 101.3 mg/L, procalcitonin of 19 ng/mL. Initial chest x-ray showed a right middle lobe infiltrate (Figure 1) and computed tomography angiography (CTA) of the chest was negative for PE and demonstrated a right middle and lower lobe infiltrate with a parapneumonic effusion (Figure 2). The patient was also tested for coronavirus disease-19 (COVID-19) which was negative. The patient was initially started on ceftriaxone and azithromycin. Transthoracic echocardiogram was negative for thrombus or vegetations. On day 3, he was clinically improving, and blood cultures grew *Pasteurella multocida* that was susceptible to penicillin. The patient’s clinical status significantly improved, and laboratory findings trended down to normal limits. He was switched to amoxicillin at the time of discharge to complete a 10-day treatment course.

## Discussion

*Pasteurella multocida* is a highly versatile gram-negative coccobacillus capable of causing infections in a wide range of domestic and wild animals as well as in humans [1,5]. Infections are most commonly transmitted by dogs and cats, as 70-90% of cats and 20-50% of dogs are colonized by *Pasteurella multocida* [2,6]. In general, these animal bites or scratch wound infections account for roughly 300,000 (1%) of all emergency department visits per year in the United States [6,7]. However, PM is an opportunistic pathogen with a predilection for immunocompromised patients [7]. Most patients with PM pulmonary infections are elderly with underlying lung diseases such as chronic obstructive pulmonary disease (COPD), bronchiectasis, or malignancy [1,8]. The mortality rate of patients with PM pleuropulmonary infection may be as high as 30% [1]. Pasteurella infections should be considered in those who have recurrent animal exposures such as veterinarians, butchers, animal breeders, farm and zoo workers. Moreover, pet owners are at increased risk of carriage or infection [2]. This organism grows in various commercial mediums, sheep blood being the preferred culture medium, followed by chocolate agar, Mueller-Hinton agar but not in MacConkey agar [2]. Regarding treatment, penicillin was found to be effective, and the treatment duration depends on disease severity, and antibiotic duration for 10-14 days is recommended [1]. Moreover, close contact with those animals should be done with caution and should be avoided if possible.

## Conclusions

PM is a rare cause of pneumonia; however, early detection and treatment is necessary. Understanding the range of presentations of PM pneumonia, including those who present with hemoptysis and in those who are immunocompetent, is necessary in order to keep a wide spectrum of differential diagnoses. Moreover, obtaining a detailed patient history about animal exposure is of paramount importance for the diagnosis of PM infections.
